# Disruption of *C. elegans* embryonic P granules upon *dlc-1(RNAi)* is not associated with P granule component loss

**DOI:** 10.17912/micropub.biology.000700

**Published:** 2022-12-06

**Authors:** Mary Ellenbecker, Ekaterina Voronina

**Affiliations:** 1 University of Montana

## Abstract

Dynein light chain (DLC-1) is a light chain component of the dynein motor complex, it functions as an allosteric regulator of multi-subunit protein complexes and promotes P granule integrity in the *C. elegans* embryo. P granules are RNA-protein complexes located in the *C. elegans* germline that are important for RNA regulation and fertility. To further study the role of DLC-1 during *C. elegans* embryogenesis we performed quantitative tandem mass tag mass spectrometry on embryos after *dlc-1* knock down. The amount of core P granule components and nucleoporin proteins did not change after *dlc-1(RNAi).* These results show that DLC-1 does not help regulate P granule protein levels and support the model that DLC-1 facilitates phase separation of P granule components *in vivo* .

**Figure 1. Mass spectrometric analysis of C. elegans embryo after dlc-1 knock down:**
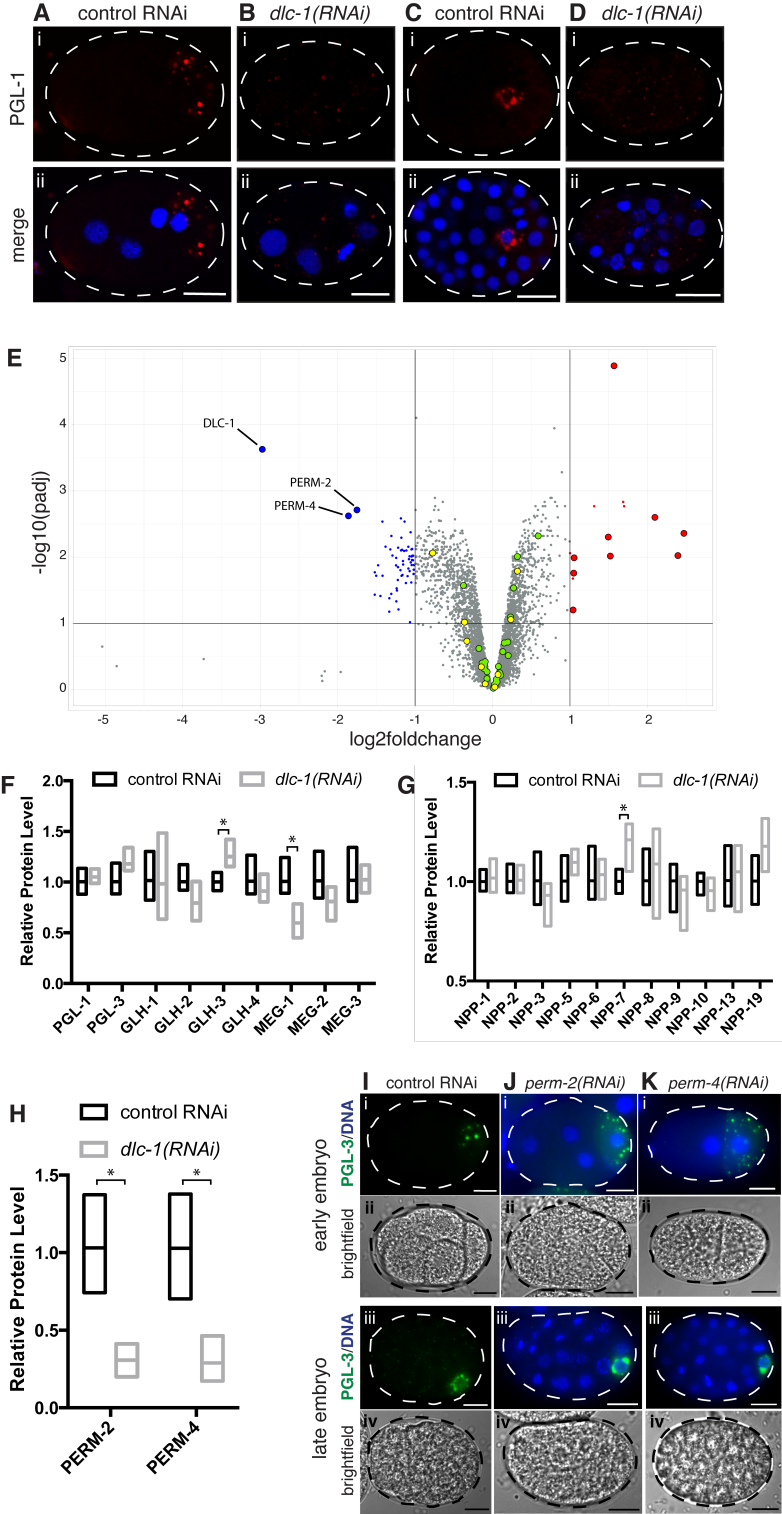
A-D) Immunostaining of control or *dlc-1(RNAi)* embryos. P granule protein PGL-1 is red and DNA is labeled with DAPI (blue). (A, B) 4 cell embryos (C, D) ~40 cell embryos. Images were acquired using a confocal microscope. Scale bars are 10 μM. Dotted lines outline embryos, which are oriented with anterior to the left and posterior to the right in this and subsequent figures. P granules are larger and restricted to the posterior cell of the 4 cell embryo and perinuclear in the 40 cell embryo in control RNAi (A, C) but mis-localized, smaller and dispersed after *dlc-1(RNAi)* (B, D). E) Volcano plot of mass spectrometric analysis of control RNAi vs. *dlc-1(RNAi)* treated *C. elegans* embryos. ‑log _10_ (Padj) (y-axis) is plotted against the average log _2_ fold change in protein level from 5 biological replicates (x-axis). Vertical black lines denote log _2_ -fold change of -1 and 1 while the horizontal black line indicates an adjusted P-value of 0.01. Blue or red color indicates a significant fold change: blue are significantly decreased 2 fold or more; *Padj* <0.01, red are significantly increased 2 fold or more; *Padj* <0.01. As expected, DLC-1 protein levels are significantly decreased in *dlc-1(RNAi)* embryos and the PERM proteins were chosen for follow up analysis (enlarged blue points). Enlarged red points correspond to significantly increased proteins involved in stress response pathways, enlarged yellow points are P granule proteins and the enlarged green points are nucleoporin proteins. F) Relative protein levels of the core P granule proteins (corresponding to enlarged yellow dots in Figure 1E). *dlc-1(RNAi)* values were scaled to the mean of the control (from 5 replicate values), which was set to 1. *- *Padj* <0.01. G) Relative protein levels of the nucleoporin proteins previously shown to be important for P granule integrity (Voronina and Seydoux 2010). *- *Padj* <0.01. H) Relative protein levels of PERM-1/2 proteins. *-Padj<0.01. I-K) Live imaging of control RNAi, *perm-2(RNAi)* or *perm-4(RNAi)* treated embryos. (Ii and Iii-Ki and Kii) 2-4 cell embryos. (Iiii and Iiv-Kiii and Kiv) ~40 cell embryos. (Ii and Iiii -Ki and Kiii) White dashed lines outline embryos, GFP-tagged P granule protein PGL-3 is green and DNA is labeled with Hoechst 33342 (blue). Scale bars are 10 μM. (Iii and Iiv-Kii and Kiv) Brightfield images of embryos outlined by black dashed lines. Scale bars are 10 μM. Notice that Hoechst 33342 is able to penetrate the nematode eggshell only after *perm-2* or *perm-4* knockdown (compare Ii to Ji and Ki and Iiii to Jiii and Kiii).

## Description

Germ granules are membraneless organelles composed of a heterogenous mixture of RNA and protein that are important for RNA regulation, germ cell development and fertility. In the model organism *Caenorhabditis elegans* , germ granules are called P granules and are present throughout the *C. elegans* life cycle. During embryogenesis P granules are highly dynamic. During the first four cell divisions P granules are cytoplasmic as they segregate asymmetrically with the P cell lineage that produces the primordial germ cells but then surround the nucleus of the P3 cell at the ~16 cell stage (Strome and Wood 1982; Figure 1A, C). At the ~100 cell stage the P4 cell divides producing the Z2 and Z3 cells, which are the founder cells for the *C. elegans* germline. Genetic analysis uncovered a number of core P granule components required for formation of these organelles (reviewed in Voronina 2013). Recently we identified dynein light chain (DLC-1) as an additional important contributor to P granule integrity in the *C. elegans* embryo as loss of *dlc-1* disrupts embryonic P granules *in vivo* (Figure 1A-D) and (Day et al. 2022). DLC-1 is a light chain component of the dynein motor complex and also functions as an allosteric regulator by promoting dimerization and structural stabilization of other multivalent protein complexes.

Additional genes important for P granule integrity include nucleoporins. The nuclear pore complex (NPC) is a large multi-subunit protein complex (~50 MDa and composed of ~30 nucleoporin proteins) that regulates molecular traffic between the cytoplasm and the nucleus. It is a cylindrical transmembrane ring structure that connects the nucleoplasm to the cytoplasm. Previous research has shown that a subset of nucleoporin proteins colocalize with both cytoplasmic and perinuclear P granules and are required for P granule integrity (Sheth et al. 2010; Updike et al. 2011; Updike and Strome 2009; Voronina and Seydoux 2010). The link between nuclear transport and P granules may be important for post-transcriptional mRNA regulation during development.

Although we know that DLC-1 is important for P granule integrity, one outstanding question is whether the apparent dispersal of P granules in the absence of DLC-1 is due to a decrease in the levels of core P granule proteins or nuclear pore complex components. We previously confirmed that *dlc-1* knock down did not impact the levels of the P granule components PGL-1 and PGL-3, but did not assess the levels of other P granule or nuclear pore complex components (Day et al., 2022). Since a candidate-based testing approach is inefficient, we used a tandem mass tag (TMT) mass spectrometry to perform a global proteomic analysis of protein levels in *dlc-1(RNAi)* vs. control RNAi treated *C. elegans* embryos.

We confirmed that the RNAi-mediated knockdown of *dlc-1* was effective and decreased DLC-1 protein levels ~8-fold (Figure 1E). The level of P granule components PGL-1, PGL-3, GLH-1, -2, -4 and MEG-2, -3 were not significantly decreased in *dlc-1(RNAi)* treated embryos compared to control. GLH-3 was significantly increased ( *Padj* <0.016) and MEG-1 was significantly decreased ( *Padj* <0.0087), however, the changes were less than 2-fold (Figure 1E, F). The reduction in MEG-1 levels upon *dlc-1(RNAi)* is consistent with our previous observations in the oocytes (Day et al. 2018). MEG-4, another core P granule component, was not detected by our analysis. The level of NPP-21 showed a small but significant decrease after *dlc-1* knock down (log2FC -0.38; *Padj* <0.027), however, the subset of nucleoporin proteins that previously exhibited a disrupted P granule phenotype when knocked down (Voronina and Seydoux 2010) showed no significant decrease in *dlc-1(RNAi)* vs. control RNAi treated embryos (Figure 1E, G). The remaining nucleoporin proteins (NPP-4, -11, -12, 14-18, -20, 22-25) and MEL-28, which is involved in nuclear pore complex assembly, did not show a significant decrease in levels after *dlc-1(RNAi)* when compared to control RNAi treated embryos (Figure 1E). Since mass spectrometric analysis of PGL-1 and PGL-3 in control vs. *dlc-1(RNAi)* treated embryos matches the PGL levels previously obtained by Western blot (Day et al. 2022), this result serves as a control for the current assay and also emphasizes the robustness of the TMT mass spectrometry method for reliably quantitating a large number of proteins from an organism. Overall, we concluded that P granule dispersal upon *dlc-1(RNAi)* was not associated with a loss of any core components or nucleoporins.

Analysis of the proteins significantly affected by *dlc-1* knockdown suggested that many of the significantly increased proteins (9/14) are related to cellular response to pathogen or stress (Figure 1E). This result might reflect cellular stress upon *dlc-1(RNAi)* associated with 55-100% embryonic lethality and reinforces the idea that DLC-1 plays an important role during embryogenesis. We then considered whether downregulation of specific proteins upon *dlc-1(RNAi)* might indirectly disrupt P granules. Two proteins that were affected the strongest were PERM proteins, PERM-2 and PERM-4, which showed a -1.7 and -1.8 log2 fold change in *dlc-1(RNAi)* treated embryos compared to control, respectively (Figure 1E, H). PERM proteins are components of the vitelline layer that is important for the structural integrity and impermeability of the nematode eggshell (González et al. 2018). PERM-2/PERM-4 localization is mutually dependent, such that a loss of one component leads to the absence of the other one in the vitelline layer (González et al. 2018). Knockdown of either *perm-2* or *perm-4* using RNA interference caused embryos to become permeable to a small molecule (Hoechst 33342 dye) that binds DNA but did not disrupt P granules (Figure 1I-K). This result suggests that although the PERM proteins are decreased significantly after *dlc-1* knockdown, they are not required for P granule integrity.

Although in some instances DLC-1 is involved in post-transcriptional gene regulation through its interaction with RNA-binding proteins such as GLD-1 and FBF-2 (Ellenbecker et al. 2019; Wang et al. 2016), our mass-spectrometry analysis suggests that DLC-1 does not promote accumulation of P granule or nucleoporin proteins. These data provide additional support for our current model that DLC-1 is involved in protein-protein interactions that facilitate P granule assembly (Day et al. 2022).

## Methods

***C. elegans* strain maintenance**

*C. elegans* strain UMT77 (Day et al. 2022) expressing GFP-tagged PGL-3 transgene ( *unc119(ed3); mntIs9 [GFP::PGL-3]* ) was maintained on new nematode growth media (NNGM) plates seeded with *E. coli* strain OP50 and maintained at 24°C (Brenner 1974). Prior to treatment with RNA interference, worms were synchronized as L1 larvae by dissolving gravid hermaphrodites in bleach (0.6% NaOCl, 1 M KOH) and hatching recovered embryos in M9 buffer (22 mM KH _2_ PO _4_ , 22 mM Na _2_ HPO _4_ , 85 mM NaCl, 1 mM MgSO _4_ ) overnight at 20°C.

**RNA interference**

RNAi plasmid targeting *perm-4* was obtained from the Source BioScience RNAi library (Kamath et al. 2003) and RNAi plasmids targeting *dlc-1* and *perm-2* were generated by PCR amplification and cloning of genomic sequences into the L4440 vector. The identity of all RNAi constructs was verified by sequencing. Plasmid DNA was transformed into HT115 *E. coli* and bacterial colonies were cultured in LB/100 μg/mL carbenicillin broth for 4 hours and then expression of double stranded RNA was induced with 10 mM isopropyl β-D-1-thiogalactopyranoside (IPTG) for either 1h ( *perm-2* and *-4* ) or 2h ( *dlc-1* ) at 37°C. After induction bacterial cells were pelleted and seeded onto NNGM plates containing 100 μg/mL carbenicillin and 0.4 mM IPTG. *C. elegans* were fed HT115 *E. coli* expressing double-stranded RNA targeting *dlc-1, perm-2, perm-4* or L4440 empty vector control starting at L1 larval stage for 96 hours at 20°C. The efficiency of the *dlc-1* RNAi treatments was established by confirming 55-100% embryonic lethality. Efficacy of *perm* RNAi treatments was confirmed by assessing increased permeability of the nematode eggshell to a small molecule (Hoechst 33342 dye).

**Protein sample collection for mass spectrometry**

*C. elegans* embryos were harvested for mass spectrometry from synchronous cultures of adult worms using bleach to dissolve the worm body. Protein was released from embryos by sonication in buffer containing 50 mM HEPES pH 7.4, 1 mM EGTA, 3 mM MgCl _2_ , 150 mM KCl, 10% glycerol and 0.05% NP-40. Protease inhibitor cocktail (Roche; 1 tablet per 6-9 mL) and 1 mM phenylmethylsulfonyl fluoride (PMSF) were added to buffer immediately before protein extraction via sonication. After sonication, embryo lysates were centrifuged for 30 minutes to remove cellular debris and total protein was measured from the extract using a Qbit protein assay kit (Life Technologies). Our protocol consistently yielded several hundred micrograms of high-quality total protein from *C. elegans* embryos.

**Mass spectrometry: CME bHPLC TMT methods – Orbitrap Eclipse**

Mass spectrometry was performed at the IDeA National Resource for Quantitative Proteomics using methods previously described in (Matalkah et al. 2022) and recapitulated here. Proteins were reduced, alkylated, and purified by chloroform/methanol extraction prior to digestion with sequencing grade modified porcine trypsin (Promega). Tryptic peptides were labeled using tandem mass tag isobaric labeling reagents (ThermoFisher Scientific) following the manufacturer’s instructions and combined into one 10-plex sample group. The labeled peptide multiplex was separated into 46 fractions on a 100 x 1.0 mm Acquity BEH C18 column (Waters) using an UltiMate 3000 UHPLC system (ThermoFisher Scientific) with a 50 min gradient from 99:1 to 60:40 buffer A:B ratio under basic pH conditions, and then consolidated into 18 super-fractions. Each super-fraction was then further separated by reverse phase XSelect CSH C18 2.5 um resin (Waters) on an in-line 150 x 0.075 mm column using an UltiMate 3000 RSLCnano system (ThermoFisher Scientific). Peptides were eluted using a 75 min gradient from 98:2 to 60:40 buffer A:B ratio. Eluted peptides were ionized by electrospray (2.4 kV) followed by mass spectrometric analysis on an Orbitrap Eclipse Tribrid mass spectrometer (ThermoFisher Scientific) using multi-notch MS3 parameters. MS data were acquired using the FTMS analyzer in top-speed profile mode at a resolution of 120,000 over a range of 375 to 1500 m/z. Following CID activation with normalized collision energy of 35.0, MS/MS data were acquired using the ion trap analyzer in centroid mode and normal mass range. Using synchronous precursor selection, up to 10 MS/MS precursors were selected for HCD activation with normalized collision energy of 65.0, followed by acquisition of MS3 reporter ion data using the FTMS analyzer in profile mode at a resolution of 50,000 over a range of 100-500 m/z. Buffer A = 0.1% formic acid, 0.5% acetonitrile. Buffer B = 0.1% formic acid, 99.9% acetonitrile. Both buffers were adjusted to pH 10 with ammonium hydroxide for offline separation.

**Mass spectrometry data analysis**

Analysis of mass spectrometry data was also performed at the IDeA National Resource for Quantitative Proteomics using methods previously described in (Urena et al. 2022) and recapitulated here. Briefly, proteins were identified and MS3 reporter ions quantified using MaxQuant (Max Planck Institute) against the UniprotKB *Caenorhabditis elegans* database (March 2021) with a parent ion tolerance of 3 ppm, a fragment ion tolerance of 0.5 Da, and a reporter ion tolerance of 0.003 Da. Scaffold Q+S (Proteome Software) was used to verify MS/MS based peptide and protein identifications. Protein identifications were accepted if they could be established with less than 1.0% false discovery and contained at least 2 identified peptides; protein probabilities were assigned by the Protein Prophet algorithm (Nesvizhskii et al. 2003) and to perform reporter ion-based statistical analysis.

Protein TMT MS3 reporter ion intensity values are assessed for quality using ProteiNorm (Graw et al. 2020). The data was normalized using cyclic loess and statistical analysis was performed using Linear Models for Microarray Data (limma) with empirical Bayes (eBayes) smoothing to the standard errors (Ritchie et al. 2015). Proteins with an FDR adjusted p-value < 0.05 and a fold change > 2 are considered to be significant.

**Immunostaining and confocal microscopy**

Gravid hermaphrodites were dissected to release embryos on slides treated with poly-L-lysine. Next, samples were frozen on dry ice, fixed in 100% methanol at -20 ^o^ C for 1 min, followed by fixation in 2% paraformaldehyde/100 mM K _2_ HP0 _4_ (pH7.2) for 5 min at room temperature. Samples were blocked in PBS/0.1% BSA/0.1% Tween-20 (PBS-T/BSA) for 30 min at room temperature and then incubated with primary antibody diluted in PBS-T/BSA overnight at 4°C. Next day, slides were washed 3x 10 min in PBS-T/BSA and then incubated with secondary antibody diluted in PBS-T/BSA at room temperature for 2 hours. After incubation with secondary antibody slides were washed again 3x 10 min using PBS-T/BSA and then 10 uL Vectashield with DAPI (Vector Laboratories, Burlingame, CA) was added to each sample before cover-slipping. Primary antibodies were rabbit anti-GFP (1:200; Life Technologies) and mouse anti-PGL-1 (5.2 ug/mL 1:125, K76; Developmental Studies Hybridoma Bank). Secondary antibodies were Alexa Fluor 488-conjugated goat anti-rabbit (1:200; Jackson ImmunoResearch) and Alexa Fluor 594-conjugated goat anti-mouse IgM (1:200; Jackson ImmunoResearch). Images of embryos were acquired using a Zeiss LSM 880 confocal microscope.

**Live imaging microscopy**

Either *perm-2(RNAi)* , *perm-4(RNAi)* or control RNAi treated gravid hermaphrodites were washed in M9/10 μg/mL Hoechst 33342 dye and dissected to release embryos. Samples were cover-slipped and incubated for 5 minutes before imaging. Fluorescent images were acquired using a Leica DFC300G camera attached to a Leica DM5500B microscope. Images were processed using Adobe Photoshop CS3.
